# Human papillomavirus (HPV) genotype distribution in Malaysia: A systematic review

**DOI:** 10.1186/s12879-025-11441-0

**Published:** 2025-08-10

**Authors:** Cassandra Sheau Mei Chee, Shirley Siang Ning Tan, Pei Jye Voon, Yolanda Augustin, Sanjeev Krishna, Nafeesa Mat Ali, Izzati Binti Wan Maharuddin, Xun Ting Tiong, Nur Khairiyah Binti Abdul Rahim, Adam Malik Ismail, Edmund Ui-Hang Sim, Paul CS Divis, Timothy Adrian Jinam, Melissa Siaw Han Lim

**Affiliations:** 1 Faculty of Medicine and Health Sciences, Universiti Malaysia Sarawak (UNIMAS), Kota Samarahan, Sarawak Malaysia; 2 Clinical Research Centre, Institute for Clinical Research, Sarawak General Hospital, Ministry of Health, National Institutes of Health, Kuching, Sarawak Malaysia; 3 Department of Pharmacy, Sarawak General Hospital, Ministry of Health, Kuching, Sarawak Malaysia; 4 Department of Radiotherapy, Oncology and Palliative Care, Sarawak General Hospital, Ministry of Health, Kuching, Sarawak Malaysia; 5 Department of Gynaecology, Sarawak General Hospital, Ministry of Health, Kuching, Sarawak Malaysia; 6 Department of Pathology, Sarawak General Hospital, Ministry of Health, Kuching, Sarawak Malaysia; 7 Clinical Academic Group in Institute for Infection & Immunity, City St George’s University of London, London, UK; 8 Biotechnology Programme, Faculty of Resource Science and Technology, Universiti Malaysia Sarawak (UNIMAS), Kota Samarahan, Sarawak Malaysia; 9 Malaria Research Centre, Faculty of Medicine and Health Sciences, Universiti Malaysia Sarawak (UNIMAS), Kota Samarahan, Sarawak Malaysia; 10 Institut Für Tropenmedizin, Eberhard Karls Universität Tübingen, and German Center for Infection Research (Dzif), Tübingen, Germany

**Keywords:** Human papillomavirus (HPV), Systematic review, Malaysia, Genotype

## Abstract

**Background:**

Human papillomavirus (HPV) is a key etiological factor in cervical cancer in both Malaysia and globally. It continues to pose a significant public health challenge. This systematic review aims to delineate the distribution of HPV genotypes across different demographics in Malaysia to inform targeted prevention strategies.

**Methods:**

We conducted a systematic review following PRISMA guidelines, analyzing observational studies published from 2000 onward that reported HPV genotypes in cervicovaginal samples from Malaysian women. The review utilized PubMed, SCOPUS, The Cochrane Library, APA PsycNet, and Google Scholar for literature searches, focusing on studies that employed molecular methods for HPV genotyping. Two reviewers independently screened the articles, extracted data, and assessed study quality using the Newcastle-Ottawa Scale (NOS). A descriptive analysis was performed, and findings were synthesized by genotype, region, and ethnicity.

**Results:**

The review included 22 studies from an initial pool of 2,547 articles, encompassing 44,251 women. These studies reported a HPV prevalence of up to 100% in confirmed cervical cancer cases and in general screenings from 4.5 to 47.7%. A total of 28 different HPV genotypes (high- and low-risk) were identified, with HPV16, HPV18, HPV58, HPV52, and HPV33 being the most prevalent high-risk genotypes. Genotype distributions showed significant variation across different states and ethnic groups within Malaysia, highlighting the diverse nature of HPV-related risks.

**Conclusions:**

This review provides a detailed snapshot of the HPV genotype distribution in Malaysia, underscoring the necessity for tailored public health interventions that address the regional and ethnic diversity in HPV prevalence. The findings support the need for targeted vaccination programs and enhanced screening measures to effectively combat the high rates of HPV-related (99%) cervical cancer in Malaysia.

**Supplementary Information:**

The online version contains supplementary material available at 10.1186/s12879-025-11441-0.

## Introduction

Cervical cancer is a significant cause of morbidity and mortality worldwide, ranked as the eighth most common cancer globally and fourth most prevalent cancer among women in Malaysia [1, 2]. The International Agency for Research on Cancer (2023) estimates 1,740 new diagnoses and 991 deaths annually from cervical cancer in Malaysia, where it poses a significant health burden [2]. The prevalence and distribution of human papillomavirus (HPV) genotypes have garnered increasing attention in Malaysia because of their importance in prevention strategies for cervical cancer. Although cervical cancer is effectively treatable if detected at an early stage, factors such as low socioeconomic status, lack of awareness, and limited access to early screening and treatment contribute to late detection, leading to high mortalities [3, 4].

Malaysia is a multiethnic country with a population predominantly consisting of three main ethnic groups: Malays, Chinese, and Indians. In Peninsular Malaysia, the Orang Asli represent the indigenous population and are generally divided into three major subgroups: Negrito, Senoi, and Proto-Malay. East Malaysia, in particular, is known for its rich ethnic diversity. Sabah is home to 32 distinct ethnic groups, with the Kadazan-Dusun being the largest, while Sarawak is home to 27 ethnic groups, with the Iban representing the largest. These diverse communities, especially in rural and remote areas, often face unique challenges related to healthcare access [5, 6]. The prevalence of different HPV genotypes is influenced by demographic factors such as race, age, sexual behavior, and socioeconomic status. Tan et al.. (2021) highlight issues faced by women living in rural areas where unemployment rates are notably high [7]. More than half of the women from rural areas in Malaysia have only completed primary education, resulting in limited awareness of HPV and cervical cancer [3, 7]. In high-risk populations such as young women engaged in sex work, the prevalence of cervical HPV exceeds 40%, with HPV 51 and 70 being common genotypes alongside the more established oncogenic types like HPV 16 [8]. In South Africa, multiple HPV infections are common among women with genital warts, with over 90% of cases attributed to HPV in both HIV-positive and HIV-negative individuals [9]. Limited infrastructure, language barriers, cultural beliefs, and varying levels of health literacy may hinder effective dissemination of information on HPV and cervical cancer prevention. As a result, ethnic and geographic disparities can influence HPV genotype distribution, screening uptake, and overall disease outcomes across different populations in Malaysia.

These findings highlight why targeted screening and vaccination programs should take demographic variations into account to reduce the high burden of HPV-related diseases within specific populations. In Malaysia, with its diverse demographic composition, variations in HPV genotypes can provide insights into local epidemiological trends and the effectiveness of existing healthcare interventions. Therefore, identifying the specific HPV genotypes present through extended genotyping, for example, assay reporting all 14 high-risk genotypes (HPV 16, 18, 31, 33, 35, 39, 45, 51, 52, 56, 58, 59, 66, and 68) allows for more tailored and effective clinical management of women undergoing primary HPV-based cervical cancer screening [10].

Human papillomavirus (HPV) is a small, non-enveloped DNA virus from the *Papillomaviridae* family that infects epithelial tissues, particularly in the anogenital region. More than 200 genotypes of HPV have been identified and are classified based on sequence variations in the L1 gene. These genotypes are generally grouped into low-risk (lr) types such as HPV 6 and 11, which are typically associated with benign lesions like genital warts, and high-risk (hr) types such as HPV 16 and 18, which are strongly linked to the development of cervical and other anogenital cancers. Understanding this classification is fundamental in guiding screening practices and vaccine development, as it ensures that public health strategies are aligned with the most clinically significant HPV types [11, 12].

In 2020, the World Health Organization (WHO) published a Cervical Cancer Elimination Strategy aimed at vaccinating school-aged girls and promoting screening using high-precision HPV DNA tests. By 2030, the strategy aims for 90% of girls to be vaccinated by age 15, 70% of women screened, and 90% of women with cervical abnormalities linked to care. The Ministry of Health (MOH) in Malaysia has implemented the use of Cervarix and Gardasil 4 as part of its immunization program. Currently, four HPV vaccines have WHO pre-qualification: Gardasil 9, which protects against HPV types 6, 11, 16, 18, 31, 33, 45, 52, and 58, Gardasil 4, targeting types 6,11,16, and 18, Cervarix and Cecolin-2, both of which cover HPV16 and HPV18. The efficacy and safety of HPV vaccines have been reviewed and shown to reduce the burden of cervical cancer in vaccinated populations [13]. To fulfil the WHO goal in the immunization program, a single-dose HPV vaccine, primarily focused on younger age groups, has been introduced to reduce cost and logistics issues [14]. Consequently, an effective screening strategy must incorporate the identification of a broader range of HPV types to ensure comprehensive monitoring and intervention. By targeting high-risk genotypes in both vaccination and screening measures, Malaysia can enhance its ability to reduce the incidence of cervical cancer, ultimately leading to improved health outcomes for its female population.

This review synthesizes current data on HPV genotype distribution within Malaysia, shed light on patterns that can inform clinical practices and health policies. By establishing a comprehensive overview of HPV prevalence, this study aims to support effective HPV management and prevention initiatives tailored to the Malaysian context.

### Objective

The objective of this review was to describe the genotype distribution of HPV among women with different ethnicity and geographical regions in Malaysia.

## Methods

### Study design

This systematic review was conducted following the Preferred Reporting Items for Systematic Reviews and Meta-Analyses (PRISMA) guidelines [15]. This protocol has been registered in PROSPERO (CRD420251061456). The study aimed to systematically identify and synthesize research on HPV prevalence and genotypes in Malaysia.

### Inclusion and exclusion criteria

Primary studies published from the year 2000 onwards were included to capture HPV genotype data spanning two decades of in Malaysia. This time frame aligns with key developments in HPV research, including the introduction of HPV vaccines, advancements in genotyping technologies, and the implementation of national vaccination and screening programs. Studies were included if they reported on the prevalence of HPV genotypes in Malaysia using cervicovaginal samples, regardless of the method of collection or processing. These included samples obtained via self-swabbing, clinician-collected swabs, liquid-based cytology (LBC), or formalin-fixed paraffin-embedded (FFPE) tissue, provided the sample originated from the cervix or vaginal tract. Eligible study designs included cross-sectional, case-control, and cohort studies that used molecular methods for HPV detection, such as polymerase chain reaction (PCR) or hybrid capture, and employed genotyping techniques including line probe assays, sequencing, or other validated molecular tools to determine genotype-specific distribution of high-risk (hrHPV) and/or low-risk (lrHPV) genotypes. Studies of any female population in Malaysia were eligible, including healthy women, women with abnormal cytology, and those attending screening or gynecologic services. No age restriction were applied, allowing inclusion of studies involving adolescents, adults, and older women, as long as cervicovaginal samples were used. Data disaggregated by region or ethnicity were also considered, when available, to assess geographic and racial patterns in genotype distribution. Studies were excluded if they did not report genotype-specific prevalence, did not use cervicovaginal samples, did not involve Malaysian populations, or were reviews, editorials, or conference abstracts without full data.

### Search strategy

A comprehensive literature search across four electronic databases (PubMed, Scopus, The Cochrane Library, and APA PsycNet) was conducted on 7 April 2025. To supplement this, an additional search was conducted using Google Scholar on 9 April 2025 to capture relevant grey literature and potentially overlooked studies. Keywords were identified from a preliminary review of relevant articles in the field. Articles were selected based on their relevance to the research topic, and frequently occurring keywords were extracted from their titles, abstracts, and author-assigned keywords. To ensure comprehensiveness, Medical Subject Headings (MeSH) terms in PubMed and indexed keywords in Scopus were reviewed and incorporated where relevant. Boolean operators (AND, OR) were used to refine the search strategy, optimizing sensitivity and specificity. The complete search strings used for each database are detailed in Online Appendix 1. The search strategy combined keywords and controlled vocabulary terms related to human papillomavirus (HPV), genotype prevalence, and Malaysia. No language or publication status restrictions were applied. Although the search string included terms for other Southeast Asian countries, only studies that presented primary data specific to Malaysia were included in the final review.

### Study selection

**Fig. 1:**
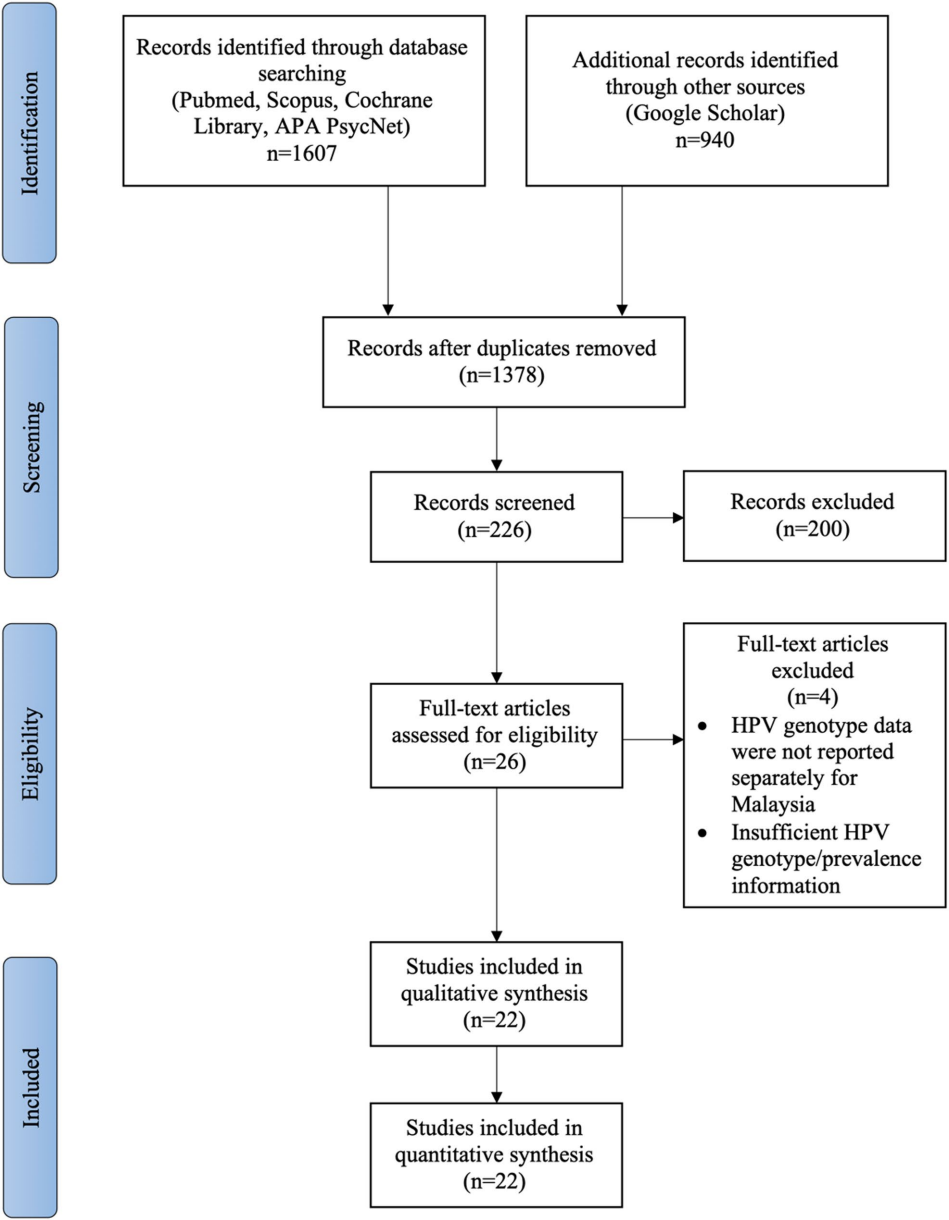
PRISMA 2020 flow diagram illustrating the study selection process. Displays the number of records identified, screened, assessed for eligibility, and included in the final review, along with reasons for exclusion at each stage.

Titles and abstracts identified through the database and grey literature searches were screened independently by two reviewers (CCSM and MLSH). Full texts of potentially relevant articles were assessed for eligibility, with discrepancies resolved through discussion or by a third reviewer (YA). The study selection process followed PRISMA 2020 guidelines (Fig. 1).

### Data extraction

Two reviewers (CCSM, MLSH) independently extracted data from each included study using a standardized data extraction form. The first reviewer has a Master of Science in Cancer Genetics, the second reviewer is a senior lecturer with a PhD in medical science specializing in women’s cancer, and the third reviewer is a clinical oncologist with a special interest in oncology and women’s cancer. Extracted information included the type of cervicovaginal sample analyzed, total sample size, geographic location of the study within Malaysia, and the HPV detection or genotyping method used. Additional variables included participant age, reported racial or ethnic background, types of high-risk (hrHPV) and low-risk (lrHPV) HPV detected, presence of single or multiple infections, and any available data regarding HPV vaccination status. Where possible, data disaggregated by geographic region and ethnicity were noted to facilitate subgroup analyses.

### Risk of bias assessment

The methodological quality of each included study was assessed using the Newcastle-Ottawa Scale (NOS) for observational studies, as described in Online Appendix 2. This tool evaluates studies across three domains: selection of study groups, comparability of groups, and ascertainment of outcomes. Risk of bias assessments were conducted independently by two reviewers (CCSM, MLSH). Disagreements were resolved through discussion, with input from a third reviewer (YA) when consensus could not be reached.

### Data analysis and synthesis

The data were analyzed descriptively. The prevalence of individual HPV genotypes was calculated using frequencies and percentages. For each study, the number of cases for specific high-risk (hrHPV) and low-risk (lrHPV) genotypes were extracted and expressed as percentages of the total sample size. Where appropriate, the frequency of single versus multiple HPV infections was also reported. In addition, regional and ethnic variations in HPV genotype prevalence were explored by grouping studies based on geographic location within Malaysia and the ethnic composition of study populations. For studies that provided sufficient data separately by ethnicity or region, the percentage prevalence of different HPV genotypes within these subgroups were calculated. All data were organized and analyzed using Microsoft Excel, in which tables and graphs were created to present the distribution of HPV genotypes and co-infections. Bar charts were generated to provide a clear visual representation of the data. Percentages, frequencies, and ranges were used to summarize the findings, facilitating comparison across studies. No meta-analysis was performed due to the variability in study designs and reporting methods. The synthesis of results was purely descriptive, and findings were summarized narratively, with emphasis on regional and ethnic differences in HPV prevalence.

## Results

### Study selection

A total of 1607 records were retrieved from database and grey literature searches: 667 from PubMed, Scopus, The Cochrane Library, and APA PsycNet (conducted on 7 April 2025), and 940 from Google Scholar (conducted on 9 April 2025). After removing 229 duplicates, 1378 records were screened by title and abstract. Of these, 1,152 were excluded for not meeting the inclusion criteria. The full texts of 226 articles were assessed, and 200 were excluded based on the review’s eligibility criteria. Four additional articles were excluded due to insufficient information, including those in which HPV genotype data were pooled with data from other countries and not reported separately for Malaysia. Ultimately, 22 studies were included in the final analysis. The study selection process is summarized in the PRISMA 2020 flow diagram (Fig. 1).

### Study characteristics

The 22 included studies were published between 2007 and 2025 and analysed cervicovaginal samples from diverse populations across Malaysia. Sample sizes ranged from 43 to 36,738 participants. Studies were conducted in multiple regions including urban and rural areas across Peninsular Malaysia and East Malaysia. Sample types varied and included liquid-based cytology (LBC), cervical swabs, self-collected vaginal samples, and paraffin-embedded cervical tissue. Participants ranged from under 20 years to over 80 years of age. Ethnicity was reported in most studies and typically included Malays, Chinese, Indians, and Indigenous populations. Detailed study information is summarized in Table 1.

**Table 1:** Characteristics of studies included in the systematic review on HPV genotype prevalence in cervicovaginal samples in Malaysia

Study (Author, Year)	Location (State/Region)	Sample Size	Sample Type	Age (range or mean ± SD)	Ethnicity/Race	HPV Genotyping Method	HPV positive (*n*)	hr-HPV detected (*n*)	lr-HPV detected (*n*)
Rahmat et al. (2021)	Kuala Lumpur, Penang, Ipoh, Johor Bahru, Kota Kinabalu	764	LBC	20–74 years	Malay, Chinese, Indian, Others, Non-Malaysians	DR.HPV Genotyping IVD kit (DR. Chip Biotechnology Incorporation, Taiwan)	107	82	25
Jailani et al. (2023)	Johor, Kedah, Kelantan, Negeri Sembilan, Selangor, Kuala Lumpur & Putrajaya	36,738	cervical swabs or self-collected samples	20–65 years	Malay, Chinese, Indian, Others	Roche Cobas 4800 HPV test (Cobas)	1666	1666	NR
Tan et al. (2018)	Kelantan, Johor Bahru	394	cervical swabs or self-collected samples	28–77 years	Malay, Chinese, Indian, Others	Hybridization Kit of GenoFlow HPV Array Test Kit (DiagCor Bioscience, Hong Kong)	154	159	8
Khoo et al. (2017)	Selangor	1293	self-sampling	18–60 years	Malay, Chinese, Indian, Others	BGISEQ-100 (Beijing Gemone Institute (BGI)-assembled Ion Proton Sequencer from Life Technologies, South San Francisco, California, USA)	86	80	6
Sainei et al. (2018)	Sabah	240	cervical swabs	21–70 years	Kadazan-Dusun, Chinese, Bajau, Sino-Native, Malay, Others	PCR (MY09/11)	24	13	11
Raub et al. (2014)	Kuala Lumpur, Selangor, Kedah, Kelantan	280	paraffin-embedded tissue biopsies	24–88 years	Malay, Chinese, Indian, Others	SACACE HPV High Risk Typing Real-TM kit (SACACE, Italy, Catalog No. TV26-100FRT)	259	488	NR
Chong et al. (2010)	Selangor	200	cervical swabs	19–60 years	Malay, Chinese, Indian	Nested PCR (MY09/MY11, GP5+/GP6+ )	84	83	1
Sharifah et al. (2019)	Kuala Lumpur	48	cervical smears	NR	NR	PCR (SPF1/SPF2)	38	36	2
Othman & Othman (2014)	Kelantan, Terengganu	635	cervical swabs	43 ± 10.5 years	Malay, Chinese, Indian, Others	Nested PCR (MY09/MY11, GP5+/GP6+ )	28	18	3
Yi et al. (2021)	Sarawak	56	LBC	23–56 years	Malay, Chinese, Bidayuh, Iban	Nested PCR (MY09/MY11, GP5+/GP6+ )	20	17	3
Zin et al. (2023)	Kelantan	789	Extracted secondary data (women attending cervical cancer screening)	30–49 years	Malay (778), Non-Malay (11)	NR	66	66	NR
Cheah et al. (2011)	Kuala Lumpur	162	FF and FP tumor tissue	22–83 years	Malay, Chinese, Indian, Other	PCR using MY09/11 and type-specific primers	44	43	1
Jerip et al. (2025)	Sarawak	151	self-sampling	24–64 years	NR	Any- plexTM II HPV HR Detection (Seegene, South Korea)	72	72	NR
Quek et al. (2013)	Malaysia	174	FFPE tissue	53.72 ± 12.87	NR	PCR using HPV short PCR fragment/line probe assay (SPF10 PCR/LiPA25; version 1.0) system	167	149	1
Latiff et al. (2015)	Jempol, Negeri Sembilan	486	cervical swabs	20–70 years	Malay, Chinese, Indian	Nested PCR (MY09/MY11, GP5+/GP6+ )	62	54	8
Khoo et al. (2022)	Selangor	1149	self-sampling	18–45 years	Malay, Chinese, Indian, Other	BGISEQ-100 (Beijing Gemone Institute (BGI)-assembled Ion Proton Sequencer from Life Technologies, South San Francisco, California, USA)	107	124	9
Ibrahim, Z (2015)	Jempol, Negeri Sembilan	226	cervical swabs	NR	NR	Nested PCR (MY09/MY11, GP5+/GP6+ )	61	NR	NR
Tan et al. (2020)	Serian, Sarawak	43	cervical swabs	20–69 years	NR	Nested PCR (MY09/MY11, GP5+/GP6+ )	5	4	1
Jerip et al. (2020)	Bario, Sarawak	75	cervical swabs	27–83 years	Kelabit, Indonesian, Lun Bawang, Penan, Iban	Nested PCR (MY09/MY11, GP5+/GP6+ )	6	6	NR
Wan Puteh et al. (2011)	Kuala Lumpur	81	paraffin-embedded cervical tissues	30–80 years	Malay, Chinese, Indian, Others	real time PCR	75	75	NR
Asyikin, N. (2009)	Selangor	200	cervical swabs	NR	NR	Nested PCR (MY09/MY11, GP5+/GP6+ )	84	83	1
Kamaluddin, N. R. (2007)	Kuala Lumpur	67	paraffin-embedded cervical tissues	NR	NR	real time PCR	57	67	NR

This table summarizes study details including geographic location, sample size, type of cervicovaginal specimen, participant age and ethnicity, HPV genotyping method used, and the number of HPV-positive cases, classified into high-risk and low-risk types where available

### Demographic and clinical characteristics (*n* = 3,272)

The majority of participants were aged 30–39 years (*n* = 1,148, 46.0%), followed by 40–49 years (*n* = 741, 29.7%). Ethnically, Malays constituted the largest group (*n* = 1,413, 61.2%), followed by Chinese (*n* = 508, 22.0%), Indians (*n* = 278, 12.0%), and various Indigenous groups. Geographically, Sarawak contributed the highest proportion of HPV-positive cases (*n* = 103, 31.7%), followed by Sabah (*n* = 48, 14.8%) and Ipoh (*n* = 56, 16.1%). Despite its large sample size, Kuala Lumpur (*n* = 1,303) accounted for only 7.2% of HPV-positive cases. Cytological assessments showed that nearly half of the samples were classified as negative for intraepithelial lesion or malignancy (NILM) (*n* = 100, 47.6%), while a notable proportion exhibited abnormalities including low-grade squamous intraepithelial lesion (LSIL) (*n* = 48, 22.9%), high-grade squamous intraepithelial lesion (HSIL) (*n* = 12, 5.7%), and squamous cell carcinoma (SCC) (*n* = 11, 5.2%). Histology data showed a predominance of SCC (*n* = 470, 67.4%) and adenocarcinoma (ADC) (*n* = 138, 19.8%) and other types (*n* = 89, 12.8%). These demographic and clinical details are presented in Table 2.

**Table 2:** Demographic and clinical characteristics of HPV-positive individuals

Characteristics		Frequency	Percentage (%)
Age group (*n* = 2494)			
	< 20	30	1.2
	20–29	96	3.8
	30–39	1,148	46.0
	40–49	741	29.7
	50–59	253	10.1
	60–69	144	5.8
	70+	82	3.3
State (*n* = 2664)			
	Ipoh	56	16.1
	Kuala Lumpur	1303	7.2
	Johor	86	8.0
	Kedah	266	2.8
	Kelantan	116	3.6
	Negeri Sembilan	178	9.1
	Selangor	508	6.5
	Sarawak	103	31.7
	Sabah	48	14.8
Race (*n* = 2308)			
	Malay	1,413	61.2
	Chinese	508	22.0
	Indian	278	12.0
	Kadazan-Dusun	14	0.6
	Sino-Native	3	0.1
	Bidayuh	5	0.2
	Iban	7	0.3
	Kelabit	5	0.2
	Others	63	2.7
	Non-Malaysian	12	0.5
Cytology Result (*n* = 210)			
	NILM	100	47.6
	AS/AG-CUS	18	8.6
	ASC-H	1	0.5
	LSIL	48	22.9
	HSIL	12	5.7
	SCC	11	5.2
	ADC	2	1.0
	Unsatisfactory	3	1.4
	WNL	12	5.7
	ICC	3	1.4
Histology Result (*n* = 697)			
	SCC	470	67.4
	ADC	138	19.8
	ASC	5	0.7
	CIN2/3 /AIS	73	10.5
	Others	11	1.6

Values are shown as n (%), representing proportions among HPV-positive individuals with available data for each category

*NILM* Negative for Intraepithelial Lesion or Malignancy, *ASCUS* Atypical Squamous Cells of Undetermined Significance, *ASC-H* Atypical Squamous Cells, Cannot Exclude HSIL, *LSIL* Low-Grade Squamous Intraepithelial Lesion, *HSIL* High-Grade Squamous Intraepithelial Lesion, *SCC* Squamous Cell Carcinoma; *ADC* Adenocarcinoma, *ASC* Atypical Squamous Cells, *AS/AG-CUS* Atypical Squamous or Glandular Cells of Undetermined Significance, *CIN2/3* Cervical Intraepithelial Neoplasia Grade 2 or 3, *AIS* Adenocarcinoma In Situ, *ICC* Invasive Cervical Cancer, *WNL* Within Normal Limits, *Unsatisfactory* Unsatisfactory Cytology Sample

### HPV genotype prevalence

**Fig. 2:**
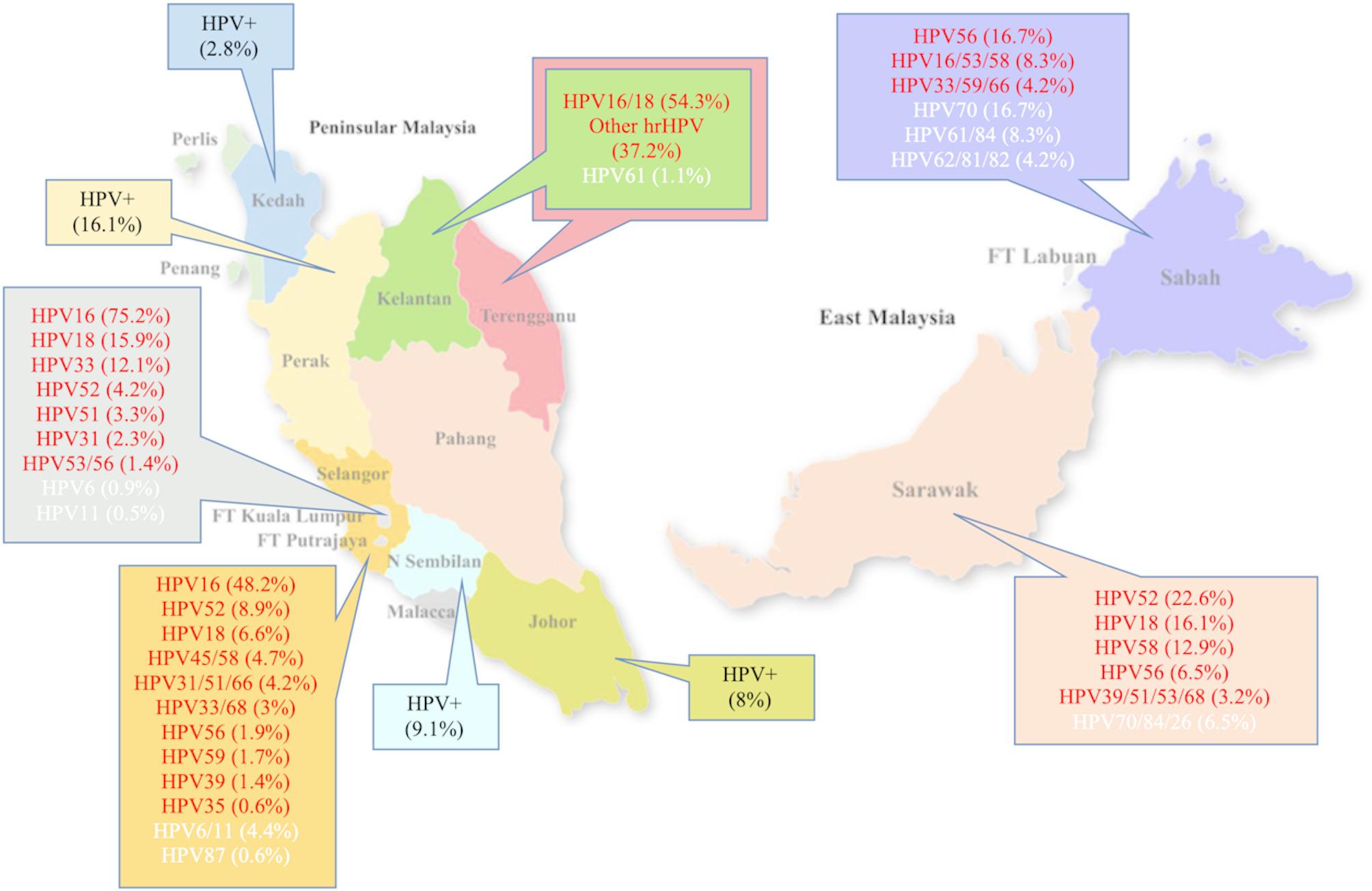
Frequency of HPV types detected in the included studies. Map shows regional HPV findings from included studies. Red indicates regions where high-risk HPV genotypes were identified, white represents low-risk HPV genotypes, and black denotes regions where HPV prevalence was reported but specific genotypes were not stated.

**Fig. 3:**
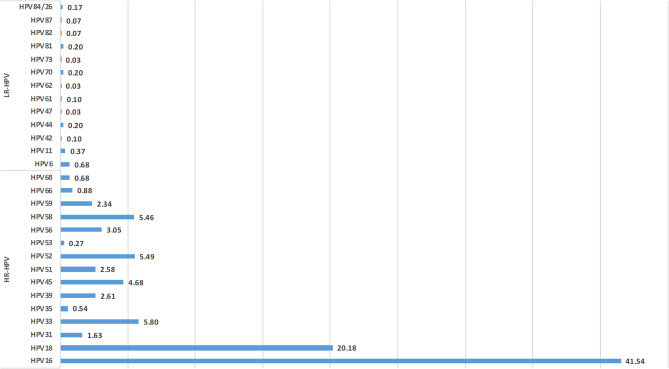
Overall distribution of HPV genotypes reported in Malaysia. A national summary of genotype prevalence across all included studies, combining high-risk and low-risk types, based on the total number of HPV-positive cases.

All included studies employed PCR-based genotyping methods, such as nested PCR, real-time PCR, and hybridization assays (e.g., SPF10/LiPA, GeneFlow Array). No studies used hybrid capture technology. The most frequently detected high-risk HPV types were HPV16, HPV18, HPV58, and HPV52, followed by HPV33 and HPV45. Low-risk types such as HPV6 and HPV11 were less commonly detected. The total frequency of HPV genotypes reported across studies is visualized in Fig. 2, which distinguishes high-risk types (red), low-risk types (white), and overall HPV-positive frequencies (black). A consolidated nationwide summary of genotype distribution is shown in Fig. 3.

### Genotype distribution by ethnicity

**Fig. 4:**
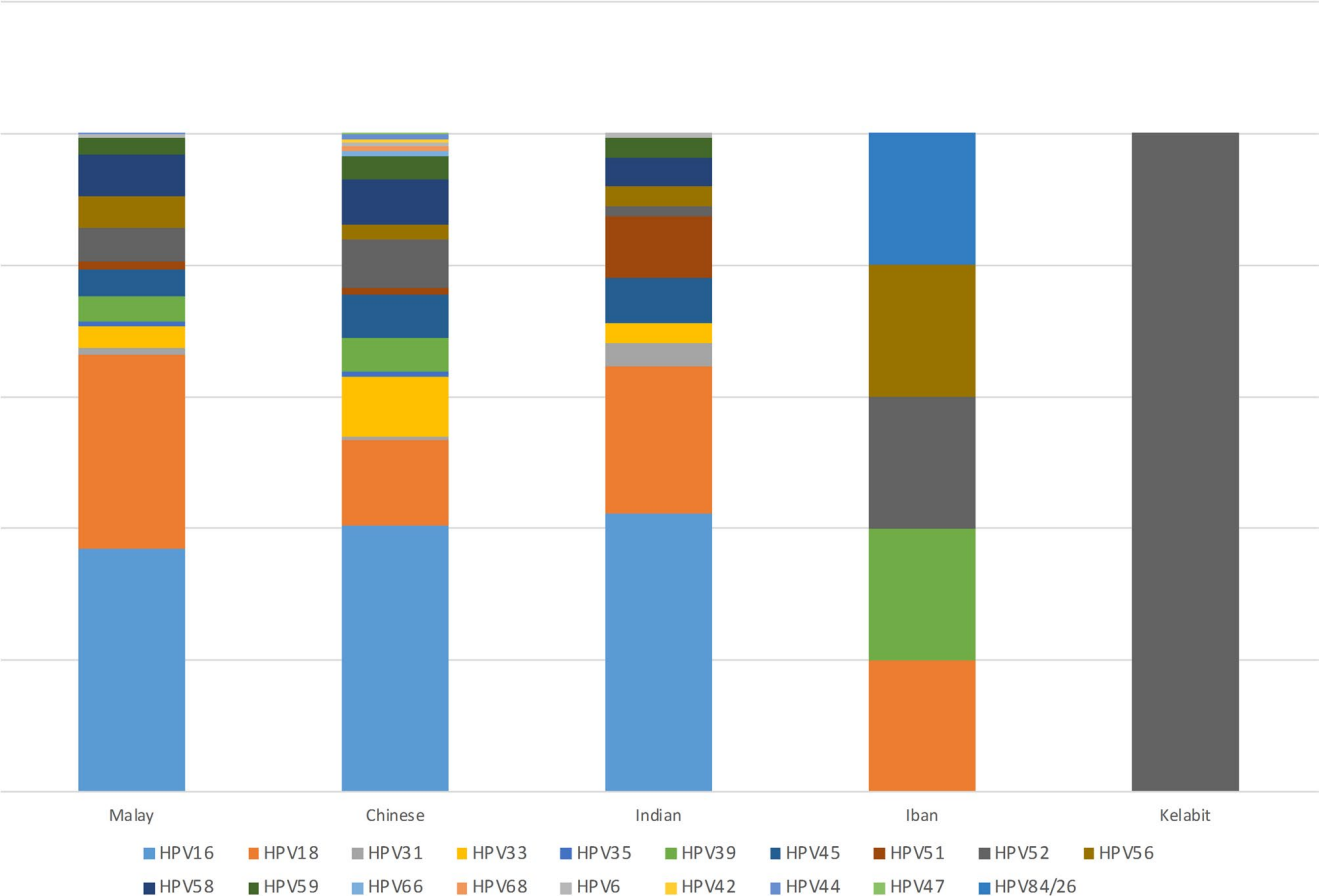
Distribution of HPV genotypes by ethnicity. Grouped bar chart comparing the prevalence of key HPV genotypes among major ethnic groups including Malays, Chinese, Indians, and Indigenous populations.

Ethnicity specific genotypic data revealed distinct trends. Among the 240 Malays, the most frequently detected genotypes were HPV16 (*n* = 192, 37.0%) and HPV18 (*n* = 153, 29.5%). In comparison, Chinese women (*n* = 200) showed a higher proportion of HPV16 (*n* = 250, 40.5%), HPV18 (*n* = 79, 12.8%) and HPV33 (*n* = 56, 9.1%), while Indian women (*n* = 180) has a notable prevalence of HPV16 (*n* = 153, 42.3%), HPV18 (*n* = 81, 22.4%), with higher rates of HPV51 (*n* = 34, 9.4%). Although data on Indigenous groups were limited, HPV52 and HPV58 appeared prominently among Iban, and 100% of detected types among Kelabit were HPV52. These patterns are visualized in Fig. 4. The total number of genotypes reported may exceed the number of individuals in each ethnic group due to multiple infections. Additionally, these data represent a subset of studies that reported genotype distribution by ethnicity, and therefore may not reflect all included studies.

### Risk of bias assessment

Study quality was assessed using the Newcastle-Ottawa Scale (NOS) for observational studies. Two reviewers (CCSM and MLSH) independently evaluated each study, with no major disagreements. Among the 22 included studies, NOS scores ranged from 6 to 9. Specifically, 3 studies scored 9, 7 studies scored 8, 10 studies scored 7, and 2 studies scored 6. Overall, most studies were of moderate to high quality.

## Discussion

Malaysia has a multi-ethnic population, and HPV prevalence varies across different races and regions. The country faces a crucial challenge in reducing HPV infections due to limited data. This systematic review provides a comprehensive overview of HPV genotype prevalence in cervicovaginal samples across Malaysia, based on 22 studies from both Peninsular and East Malaysia. The findings highlight a consistently high prevalence of high-risk HPV (hrHPV) types, particularly HPV16 and HPV18, which are the two genotypes most commonly associated with cervical cancer globally. Other frequently detected hrHPV genotypes include HPV52, HPV58, HPV33, and HPV45, which were found across various population groups and regions. These trends are consistent with previous studies in Asia and globally [16–18].

HPV infection was more commonly found in women aged 30 years and above, accounting for 75.8% of reported cases [16, 19–23]. This trend likely reflects persistent infection acquired earlier in life, combined with the fact that these cohorts were not eligible for the Malaysia’s school-based HPV immunization program, which was introduced in 2010 and targets 13-year-old girls. The publication window beginning in 2000 therefore includes women largely unvaccinated at the time of data collection. Although awareness of HPV has historically been low, modest improvements have been observed in recent years [24]. Among the general population, HPV prevalence ranged from 4.4 to 46.7%, whereas in women with abnormal smears or cervical cancer, prevalence ranged from 27.2 to 100%.

In cytologically normal samples, HPV detection was notably high (47.6%), similar to trends seen in Thailand (72.9%) [25]. Among cytological abnormalities, HPV was most prevalent in LSIL (22.9%), followed by atypical squamous or glandular cells (8.6%), and HSIL (5.7%). In cervical cancer specimens, HPV infection was found in 67.4% of SCC cases, 19.8% in ADC, and 0.7% in atypical squamous cells (ASC). HPV16 emerged as the most frequently detected genotype across all lesion grades, followed by HPV18, 58, 52, and 33, though distribution varied by lesion severity, ethnicity, and region [17, 22, 26, 27].

Overall, high-risk HPV types accounted for 97.7% of cases, while low-risk types made up 2.3%. Notably, the genotype distribution varied across regions. In West Malaysia, HPV16 and HPV18 were the most prevalent types. In East Malaysia, HPV52 and HPV58 were predominant in Sarawak, while HPV56 and HPV70 were more frequently observed in Sabah. Interestingly, HPV70 was more common in East Malaysia compared to the West, aligning with broader regional trends noted by Peng et al. (2012) and supporting the idea of geographic variation in genotype distribution across East and Southeast Asia [18].

This review further emphasizes variation in HPV genotype distribution among different ethnic groups. Among Malaysians, HPV prevalence was highest among Malays (61.2%), followed by Chinese (22.0%) and Indians (12.0%). In East Malaysia, specific Indigenous populations, such as the Kadazan-Dusun, Iban, and Bidayuh, also showed evidence of HPV infection, though sample sizes were limited. The distribution of genotypes also varied: HPV58, HPV52, and HPV56 were more common among Malays; HPV33, 52, and 45/58 among Chinese; and HPV51, 45, and 58 among Indians. Although research on ethnic disparities in HPV prevalence is limited in Malaysia, these findings are consistent with international data showing variation across ethnic groups. For example, HPV positivity was highest among non-Hispanic Blacks and lowest among Mexican-born Hispanic Whites in the U.S [28].

Given that HPV is responsible for over 95% of cervical cancer cases globally, these findings reinforce the critical importance of early detection and prevention [29]. Vaccination remains the most effective primary prevention method. While the bivalent vaccine provides nearly complete protection against HPV16 and 18 [3, 30, 31], the broader protection offered by the 9-valent vaccine (covering HPV31, 33, 45, 52, and 58) is particularly relevant to the Malaysian context [32]. This review found that, in addition to HPV16 and 18, high-risk types such as HPV52, 58, and 33 were frequently detected, especially in East Malaysia and among certain ethnic groups. These genotypes are included in the nonvalent vaccine, suggesting that broader adoption of this vaccine could enhance protection in regions and population where non-16/18 types are more prevalent. For instance, HPV52 and 58 were predominant in Sarawak, while HPV33 was more frequently found among Chinese women. Aligning vaccine strategy with these distribution patterns could strengthen national prevention efforts and help reduce regional and ethnic disparities in cervical cancer outcomes. Nevertheless, gaps in vaccine coverage and public access persist, especially in rural areas and among underrepresented populations [3, 33].

These findings support the ongoing need for regionally tailored HPV prevention strategies in Malaysia. This includes expanding surveillance, enhancing access to the nonavalent HPV vaccine, and integrating education and outreach programs to address persistent disparities in screening and vaccination uptake.

### Strength and limitation

This systematic review provides valuable insights for revising Malaysia’s current vaccination strategies and HPV-based screening programs. The inclusion of studies from diverse settings and populations provides a broader understanding of HPV genotype distribution across the country. However, certain limitations should be considered when interpreting the findings. First, the predominance of studies from West Malaysia restricts a comprehensive assessment of HPV genotype distribution nationwide. Second, variations in HPV genotyping methods and differences in study participant selection may contribute to inconsistencies in the reported data. Finally, the possibility of participant overlap across studies especially those using national or institutional databases. Due to limited reporting in the original studies, we could not fully verify the independence of all samples, which may introduce some duplication bias.

## Conclusions

This review provides a comprehensive analysis of HPV genotype distribution across ethnic groups and regions in Malaysia. While HPV16 and HPV18 remain the most common genotypes nationwide, regional variations are evident, such as HPV52 and HPV58 are more prevalent in Sarawak and HPV56 and HPV70 are more frequently observed in Sabah. Ethnic variation in genotype susceptibility also suggests potential genetic, behavioral, and environmental influences. Interestingly, HPV prevalence was found to be higher in women with normal cytology findings compared to those with abnormal findings, highlighting the importance of screening beyond cytological abnormalities. These findings reinforce the need for region-specific HPV vaccination approaches, such as expanding the coverage of the nonavalent vaccine in East Malaysia, where other high-risk types apart from HPV16/18 are common, and enhancing screening among women aged 30 and above, many of whom fall outside the national school-based vaccination program launched in 2010. To guide future policies, longitudinal studies are essential for tracking genotype distribution post-vaccination and updating national screening guidelines accordingly. Strengthening surveillance systems and improving data collection, especially in rural and underrepresented regions, will be critical to achieving accessible and regionally tailored HPV prevention across Malaysia.

## Supplementary Information

Below is the link to the electronic supplementary material.

Supplementary Material 1

Supplementary Material 2

## Data Availability

The dataset generated and/or analysed during the current study are available in the [HPV Systematic Review] repository, https://www.kaggle.com/datasets/melissalimsiawhan/hpv-systematic-review.

## Abbreviations

HPV: Human Papillomavirus
PRISMA: Preferred Reporting Items for Systematic Reviews and Meta-Analyses
NOS: Newcastle-Ottawa Scale
LBC: Liquid-Based Cytology
FFPE: Formalin-Fixed Paraffin-Embedded
hrHPV: High-Risk Human Papillomavirus
lrHPV: Low-Risk Human Papillomavirus
MOH: Ministry of Health
WHO: World Health Organization
SCC: Squamous Cell Carcinoma
ADC: Adenocarcinoma
NILM: Negative for Intraepithelial Lesion or Malignancy
ASCUS: Atypical Squamous Cells of Undetermined Significance
ASC-H: Atypical Squamous Cells, Cannot Exclude High-Grade Squamous Intraepithelial Lesion
LSIL: Low-Grade Squamous Intraepithelial Lesion
HSIL: High-Grade Squamous Intraepithelial Lesion
CIN2/3: Cervical Intraepithelial Neoplasia Grade 2 or 3
AIS: Adenocarcinoma In Situ
ICC: Invasive Cervical Cancer
WNL: Within Normal Limits
PCR: Polymerase Chain Reaction
MeSH: Medical Subject Headings
