# Supplementary material for: Human papillomavirus (HPV) genotype distribution in Malaysia: A systematic review
Source: BMC Infect Dis. 2025 Aug 10;25:1010. doi: 10.1186/s12879-025-11441-0 (PMC12335762; doi:10.1186/s12879-025-11441-0)
Supplement: Supplementary file 1 — Supplementary Material 1 [file 12879_2025_11441_MOESM1_ESM.docx]

Appendix 1: Search Strategy

Table A1: Database Search Strategy for Identifying Studies on HPV Prevalence in Malaysia

| **Database** | **Date of Search** | **Search String** | **Filters Applied** | **Search Results** |
| --- | --- | --- | --- | --- |
| PubMed | 12 November 2024  Updated:  7 April 2025 | (("Human papillomavirus"[MeSH Terms] OR "HPV"[Title/Abstract] OR "Human papilloma virus"[Title/Abstract]) AND ("Prevalence"[Title/Abstract] OR "HPV prevalence"[Title/Abstract] OR "HPV type prevalence"[Title/Abstract] OR "HPV infection"[Title/Abstract] OR "HPV genotyping"[Title/Abstract]) AND ("Cervical"[Title/Abstract] OR "Cervical cancer"[Title/Abstract] OR "Cervical screening"[Title/Abstract]) AND ("Malaysia"[Title/Abstract] OR "Indonesia"[Title/Abstract] OR "Vietnam"[Title/Abstract] OR "Laos"[Title/Abstract] OR "Brunei"[Title/Abstract] OR "Thailand"[Title/Abstract] OR "Myanmar"[Title/Abstract] OR "Philippines"[Title/Abstract]  OR "Cambodia"[Title/Abstract] OR "Singapore"[Title/Abstract]) AND ("HPV DNA"[Title/Abstract])) | From 2000 to 2025 | 405 |
| Scopus | 13 November 2024  Updated:  7 April 2025 | (TITLE-ABS-KEY ("Human papillomavirus" OR "HPV" OR "Human papilloma virus") AND TITLE-ABS-KEY ("Prevalence" OR "HPV prevalence" OR "HPV type prevalence" OR "HPV infection" OR "HPV genotyping") AND TITLE-ABS-KEY ("Cervical" OR "Cervical cancer" OR "Cervical screening") AND TITLE-ABS-KEY ("Malaysia" OR "Indonesia" OR "Vietnam" OR "Laos" OR "Brunei" OR "Thailand" OR "Myanmar" OR "Philippines" OR "Cambodia" OR "Singapore") AND TITLE-ABS-KEY ("HPV DNA")) | From 2000 to 2025 | 171 |
| Cochrane Library | 7 April 2025 | HPV AND prevalence OR genotype AND cervical AND Malaysia | 2000-2025 | 43 |
| APA PsycNet | 7 April 2025 | (Any Field: "human papillomavirus" OR Any Field: HPV)  AND  (Any Field: prevalence OR Any Field: "HPV prevalence" OR Any Field: "HPV infection" OR Any Field: "HPV genotyping" OR Any Field: "HPV type prevalence")  AND  (Any Field: cervical OR Any Field: "cervical cancer" OR Any Field: "cervical screening")  AND  Any Field: Malaysia  AND  Any Field: "HPV DNA" | 2000-2025 | 48 |
| Google Scholar | 9 April 2025 | human papillomavirus, hpv, prevalence, hpv type prevalence, hpv genotyping, hpv infection, cervical, cervical screening, cervical cancer, malaysia, hpv dna | 2000-2025 | 940 |
