# Supplementary material for: Human papillomavirus (HPV) genotype distribution in Malaysia: A systematic review
Source: BMC Infect Dis. 2025 Aug 10;25:1010. doi: 10.1186/s12879-025-11441-0 (PMC12335762; doi:10.1186/s12879-025-11441-0)
Supplement: Supplementary file 2 — Supplementary Material 2 [file 12879_2025_11441_MOESM2_ESM.docx]

Appendix 2: Newcastle-Ottawa Scale in both cross-sectional and cohort studies

Table A2: The Newcastle-Ottawa Scale (NOS) for assessing included studies

| Study | Selection | | Comparability | | Exposures | | Total Quality score* |
| --- | --- | --- | --- | --- | --- | --- | --- |
| Author (Year) | CCSM | MLSH | CCSM | MLSH | CCSM | MLSH |  |
| Rahmat et al. (2021) | 3 | 3 | 2 | 2 | 2 | 2 | 7 |
| Jailani et al. (2023) | 3 | 3 | 2 | 2 | 2 | 2 | 7 |
| Tan et al. (2018) | 4 | 4 | 2 | 2 | 2 | 2 | 8 |
| Khoo et al. (2017) | 3 | 3 | 2 | 2 | 3 | 3 | 8 |
| Sainei et al. (12018) | 3 | 3 | 2 | 2 | 2 | 2 | 7 |
| Raub et al. (2014) | 3 | 3 | 2 | 2 | 3 | 3 | 8 |
| Chong et al. (2010) | 3 | 3 | 2 | 2 | 2 | 2 | 7 |
| Sharifah et al. (2019) | 4 | 4 | 2 | 2 | 3 | 3 | 9 |
| Othman & Othman (2014) | 3 | 3 | 2 | 2 | 2 | 2 | 7 |
| Yi et al. (2021) | 3 | 3 | 2 | 2 | 3 | 3 | 8 |
| Zin et al. (2023) | 2 | 2 | 2 | 2 | 3 | 3 | 7 |
| Cheah et al. (2011) | 4 | 4 | 2 | 2 | 3 | 3 | 9 |
| Jerip et al. (2025) | 3 | 2 | 1 | 1 | 3 | 3 | 7 |
| Quek et al. (2013) | 3 | 3 | 2 | 2 | 2 | 3 | 8 |
| Latiff et al. (2015) | 4 | 4 | 2 | 2 | 2 | 2 | 8 |
| Khoo et al. (2022) | 4 | 4 | 2 | 2 | 3 | 3 | 9 |
| Ibrahim, Z (2015) | 4 | 4 | 1 | 1 | 1 | 1 | 6 |
| Tan et al. (2020) | 3 | 3 | 2 | 2 | 1 | 1 | 6 |
| Jerip et al. (2020) | 3 | 3 | 2 | 2 | 2 | 2 | 7 |
| Wan Puteh et al. (2011) | 3 | 3 | 2 | 2 | 3 | 3 | 8 |
| Asyikin, N. (2009) | 4 | 4 | 2 | 2 | 1 | 1 | 7 |
| Kamaluddin, N. R. (2007) | 4 | 4 | 2 | 2 | 1 | 1 | 7 |

* Each study was assigned a score ranging from 0 to 9 stars, with higher scores indicating higher study quality. Studies with 7–9 stars were classified as high quality, those with 4–6 stars as moderate quality, and those with 0–3 stars as low quality.
